# Recovery of NIS expression in thyroid cancer cells by overexpression of Pax8 gene

**DOI:** 10.1186/1471-2407-5-80

**Published:** 2005-07-19

**Authors:** Ivan Presta, Franco Arturi, Elisabetta Ferretti, Tiziana Mattei, Daniela Scarpelli, Emanuele Tosi, Angela Scipioni, Marilena Celano, Alberto Gulino, Sebastiano Filetti, Diego Russo

**Affiliations:** 1 Dipartimento di Medicina Sperimentale e Clinica "G. Salvatore" and Dipartimento di Scienze Farmacobiologiche, University of Catanzaro "Magna Graecia", Catanzaro, Italy; 2 Dipartimento di Scienze Cliniche and Dipartimento di Medicina Sperimentale e Patologia, University of Rome "La Sapienza", Rome, Italy; 3 Neuromed Institute, 86077 Pozzilli, Italy

## Abstract

**Background:**

Recovery of iodide uptake in thyroid cancer cells by means of obtaining the functional expression of the sodium/iodide symporter (NIS) represents an innovative strategy for the treatment of poorly differentiated thyroid cancer. However, the NIS gene expression alone is not always sufficient to restore radioiodine concentration ability in these tumour cells.

**Methods:**

In this study, the anaplastic thyroid carcinoma ARO cells were stably transfected with a Pax8 gene expression vector. A quantitative RT-PCR was performed to assess the thyroid specific gene expression in selected clones. The presence of NIS protein was detected by Western blot and localized by immunofluorescence. A iodide uptake assay was also performed to verify the functional effect of NIS induction and differentiation switch.

**Results:**

The clones overexpressing Pax8 showed the re-activation of several thyroid specific genes including NIS, Pendrin, Thyroglobulin, TPO and TTF1. In ARO-Pax8 clones NIS protein was also localized both in cell cytoplasm and membrane. Thus, the ability to uptake the radioiodine was partially restored, associated to a high rate of efflux. In addition, ARO cells expressing Pax8 presented a lower rate of cell growth.

**Conclusion:**

These finding demonstrate that induction of Pax8 expression may determine a re-differentiation of thyroid cancer cells, including a partial recovery of iodide uptake, fundamental requisite for a radioiodine-based therapeutic approach for thyroid tumours.

## Background

Thyroid tumours show a good long-term survival, but, when we consider the poorly differentiated histotypes and in particular the anaplastic carcinoma, the prognosis is very poor [1,2]. A major determinant of such an opposite behaviour is the lost ability to concentrate the radioiodine, used as very effective tool for diagnosis and treatment for both tumour remnants or distant metastases [3]. The loss of iodide uptake capacity in poorly differentiated thyroid carcinomas is mainly due to the reduced/lost functional expression of the sodium/iodide symporter (NIS), with a defect occurring mainly at gene expression level, but in some case involving post-transcriptional or other unknown alterations [4-7]. For this reason, many strategies have been used and are currently in progress in order to re-establish iodide uptake function by means of re-expressing the NIS in tumour cells. At present, two different strategies are based on: a. introducing NIS gene in cancer cells through a viral vector; b. stimulating endogenous NIS gene expression [5,7]. The latter approach may take advantage from the description and characterization of the human NIS gene promoter as well as studies regarding NIS expression regulation [4-7]. Among the physiological stimulators of NIS gene transcription, a pivotal role is played by the thyroid-specific transcription factor Pax8 [8-10]. This homeobox protein is able to regulate, in normal thyroid cells, the expression of many thyroid specific protein and is often reduced in thyroid tumours, especially in the less differentiated histotypes [11].

In this study we analysed the effects of Pax8 overexpression on a human thyroid anaplastic cancer cell line ARO cells. It is a widely used cell line which resembles the behaviour of poorly differentiated thyroid tumours, including a very low iodide uptake function as well as lost of NIS and Pax8 gene expression, together with several other markers of thyrocyte differentiation [12]. By restoring Pax8 expression, we observed the recovery of NIS expression, together with other markers of thyroid differentiation, associated to partial ability to uptake the radioiodine.

## Methods

### Recombinant plasmid construction

The plasmid vector pCMV and the full length cDNA fragment of Pax8 A (kindly provided by Dr. Di Lauro) [13], were cleaved by Kpn I and Eco RI. The cleaved products were ligated using T4DNA ligase into pCMV-Script cloning and expression vector (Stratagene, La Jolla, CA, USA). DH5 alpha cells were transformed using the recombinant product pCMV/Pax8 and then screened for the positive clones containing the inserted fragment by colony-PCR techniques. The positive colonies were amplified, extracted, purified and identified by endonuclease cleavage. The sequences of the inserted fragments were confirmed by automated sequencing using ABI Prism 7700 Sequence Detector (Applied Biosystems, Foster City, CA, USA).

### Cell cultures and stable transfection

ARO cells were cultured in RPMI 1640 medium with 10% foetal bovine serum (Gibco, Milano, Italy) and containing penicillin/streptomycin and amphotericin B (Sigma-Aldrich S.r.l., Milano, Italy). The non-liposomal lipid mixture FUGENE 6 (Roche diagnostics, Monza, Italy) was used for transfection. ARO cells were plated at 3 × 10^5 ^cells/60 mm culture dish 24 h prior to transfection. 2 mg of the construct and 2 μg of empty vector in 200 μl of serum free medium and 12 μl of FUGENE 6 mixture each, were used for two different transfections. After 45 min of incubation at room temperature, the transfection mixture was added to the dishes with 2 ml of fresh complete medium. The cells were grown for 24 h before adding 400 μg/ml neomycin (Sigma-Aldrich S.r.l.) for selection and after 3 days the transfected cultures were split for single clone isolation. After propagation, total RNA was extracted from 24 isolated clones for screening of Pax8 gene expression by a quantitative RT-PCR. Cells transfected with the empty vector (ARO-pCMV) were used as control. FRTL-5 and CHO cells, used as additional control, were cultured as described previously [14].

### RNA extraction, reverse transcription and quantitative PCR

Total RNA was extracted from cells with using the Qiagen RNA/DNA kit (Qiagen S.p.A, Milano, Italy), according to manufacturer's instructions. First-strand cDNA synthesis was performed using 2 μg of each RNA sample primed with random hexamers with 200 U of Superscript II reverse transcriptase (Life Technologies S.r.l., San Giuliano milanese, Milano, Italy). Quantitative PCR analysis (Q-PCR) of Pax8, NIS, Thyroperoxidase (TPO), TSH-Receptor (TSH-R), Thyroid transcription factor-1 (TTF1) and pendrin (PDS) mRNA expression was performed on cDNA samples employing the primers indicated in Table 1. Oligonucleotide primers and probe were purchased from Applied Biosystems as Assays-on-Demand Gene Expression Products for the study of Thyroglobulin (Tg), endogenous control glyceraldehyde-3-phosphate dehydrogenase (GAPDH) and β-actin were purchased from Applied Biosystems as TaqMan PDAR (VIC dye-labeled). A 25 μl reaction mixture containing 2.5 μl of cDNA template, 12.5 μl TaqMan Universal PCR master mix (Applied Biosystems) and 1.25 μl primer probe mixture was amplified using the following thermal cycler parameters: incubation at 50°C for 2 min and denaturing at 95°C for 10 min, then 40 cycles of the amplification step (denaturation at 95°C for 15 sec and annealing/extension at 60°C for 1 min). For each amplification run standard curve was generated using 6 serial dilutions of cDNA mix expressing all the genes analysed. All amplification reactions were performed in triplicate, and the averages of the threshold cycles were used to interpolate standard curves and to calculate the transcript amount in samples using SDS version 1.7a software. Levels of mRNA expression were expressed after normalization with those of two endogenous controls, GAPDH and β-actin.

### Protein extraction, western blot analysis and immunofluorescence

Total proteins were extracted from thyroid cell lines as described previously [15]. Briefly, confluent cells from 3 Petri dishes were collected and homogenized in 1 ml of buffer containing 250 mM sucrose, 10 mM HEPES-KOH (pH 7.5), 1 mM EDTA, 1 mM PMSF, 10 μg/ml leupeptin, 10 μg/ml aprotinin (Sigma-Aldrich S.r.l.). The homogenate was centrifuged at 14000 × g (4°C for 15 min) and the supernatant (which contained the whole cell lysate) was quantified spectrophotometrically using the Bradford method. Twenty micrograms of proteins were loaded on a 4–20% gradient SDS-polyacrylamide gel and subjected to electrophoresis at a constant voltage (110 V). Electroblotting to a Hybond ECL-PVDF nitrocellulose membrane (Amersham Pharmacia biotech., Milano, Italy) was performed for 2 h at 125 mA using a Mini Trans blot electroblotting system (Bio-rad Laboratories S.r.l, Milano, Italy). Blocking was done using TTBS/milk (TBS, 1% Tween 20, and 5% non fat dry milk) for 2 h at room temperature. The membrane was then incubated with a 1/500 dilution of affinity purified rabbit anti-NIS polyclonal antibody [16] or a 1/5000 dilution of mouse monoclonal anti-human beta-actin antibody, overnight at 4°C in TTBS/milk. After one 15 min and two 5 min washes in TTBS, the membrane was incubated with a 1/20000 dilution of a horse radish peroxidase conjugated anti-rabbit or anti-mouse antibody (Transduction Laboratories, Lexington, KY, USA) in TTBS/milk. After one 15 min and two 5 min washes in TTBS, the protein was visualized by an enhanced chemiluminescence Western blot detection system (ECL plus, Amersham Pharmacia biotech.).

For immunofluorescence analysis, the cells were plated on glass coverslips and fixed using PBS containing 4% paraformaldehyde for 10 min at RT. Then the fixative was aspirated and the cells washed 3 times for 5 min each with PBS. The cells were incubated with blocking buffer (PBS 1× + BSA 1%) for 15 min at RT. Immunostaining was performed using 1:600 dilution of a rabbit polyclonal antibody anti h-NIS [15] for 1 h at RT. After three 5-min washes in PBS, the cells were incubated with secondary antibody conjugate with FITC (Sigma Aldrich S.r.l.) diluted 1:160 in PBS 1×, for 30 min at RT, re-washed in PBS and incubated in Hoechst 1× for 3 min at RT. After three final PBS washes, the cells were mounted with Vectashield (Vector Laboratories, Inc. Burlingame, CA, USA).

### Iodide uptake, efflux rate and cell growth rate assay

Uptake of ^125^I was measured as previously described [17]. Briefly, cells were splitted and seeded into 12-well plates and, after aspirating the culture medium, washed with 1 ml of Hank's balanced salt solution (HBSS) (Life technologies S.r.l) supplemented with Hepes 10 mM, pH 7.3. ^125^I uptake was initiated by adding to each well 500 μl buffered HBSS containing 0.1 μCi carrier-free Na^125^I and 10 μM NaI to obtain a specific activity of 20 mCi/mmol.

In half of the wells, this assay buffer was supplemented with the NIS inhibitor KClO_4 _(10 μM) to control for specific uptake. After 40 min at 37°C in a humid atmosphere, the radioactive medium was aspirated and cells were washed with 1 ml of ice-cold HBSS. One ml of 95% ethanol was added to each well for 20 min and then transferred into vials for counting with a gamma counter. Iodide uptake was expressed as picomoles per μg of DNA. Each experiment was done at least twice in quadruplicates and the FRTL-5 cells and CHO cells were used, respectively, as positive and negative controls.

Iodide efflux in wild-type and transfected ARO cells, as well FRTL-5 cells, was evaluated as previously described by Arturi et al. [14].

For cell growth rate analysis, 10^5 ^cells were plated in 60 mm dishes. Seven hours were considered as extra time needed from cells to sediment and start to proliferate. Cells were harvested after 24, 48 and 72 hours and the number of cells was determined by Neubauer cell counting chamber.

## Results

**Figure 1:**
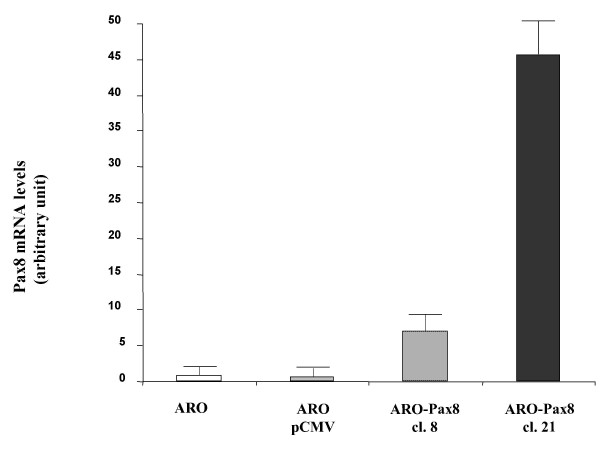
Levels of expression of Pax8 mRNA in the wild-type and transfected ARO cells. ARO cells were transfected with a pCMV expression vector empty or containing the hPax8 gene, as described in Methods. Total RNA was extracted from duplicate dishes of wild-type ARO cells (ARO), cells containing the empty vector (ARO-pCMV) and two selected clones expressing Pax8 gene (ARO-Pax8 cl. 8 and ARO-Pax8 cl. 21 (as described in Methods) and the levels of RNA were determined using the real-time PCR method. To normalize the different amount of total RNA added to the reaction, an amplification of GAPDH or β-actin was performed as endogenous control. The data are expressed as the mean ± SD of values obtained from at least three different experiments. Statistic analysis was performed using the one-factor ANOVA, followed by *t *test. *P *< 0.05 was considered statistically significant.

ARO cells were transfected with the pCMV-Script-hPax8 construct or the expression vector alone. A series of 24 clones were grown and screened for the presence of the insert (data not shown). Pax8 mRNA levels were quantified by real time RT-PCR method and ARO-Pax8 clones 8 and 21 were selected in that they overexpressed different amounts of Pax8 transcript (Fig. 1).

**Figure 2:**
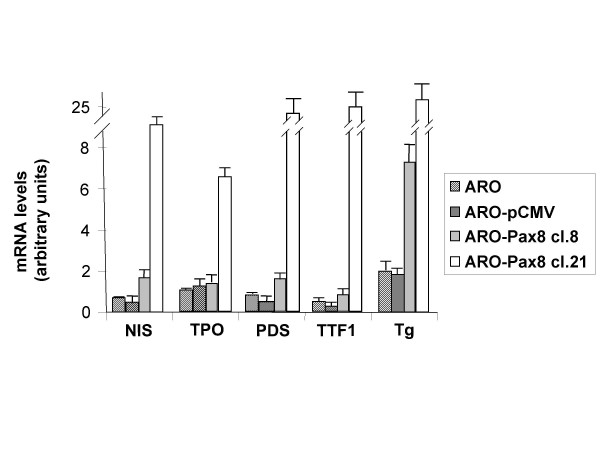
Levels of mRNA of various thyroid specific genes in wild-type and transfected ARO cells. Total RNA was extracted from duplicate dishes of wild-type ARO cells (ARO), cells containing the empty vector (ARO-pCMV), two selected clones expressing Pax8 gene (ARO-Pax8 cl. 8 and ARO-Pax8 cl. 21) and the levels of RNA were determined using the real-time PCR method. To normalize the different amount of total RNA added to the reaction, an amplification of GAPDH or β-actin was performed as endogenous control. The data are expressed as the mean ± SD of values obtained from at least three different experiments. Statistic analysis was performed using the one-factor ANOVA, followed by *t *test. *P *< 0.05 was considered statistically significant.

Since Pax8 has a key role in thyroid differentiation, we first checked in the ARO-Pax8 cells the presence of a series of thyroid specific genes. Fig. 2 shows the levels of the transcripts detected in the two clones versus wild-type ARO cells or ARO transfected with the empty vector (ARO-pCMV). The highest mRNA expression levels were detected for Thyroglobulin gene (more than 20 fold in ARO-PAX8 clone 21), but also NIS, Pendrin, TPO and TTF1 gene were expressed in PAX8 clones with different levels of the transcripts depending on the different amount of Pax8 transcript (Fig. 2). All the transcript expression levels resulted lower (ranging between 10 and 100 fold) than those detectable in normal thyroid tissue (data not shown). The mRNA of the TSH receptor did not appear in the transfected cell clones (data not shown).

**Figure 3:**
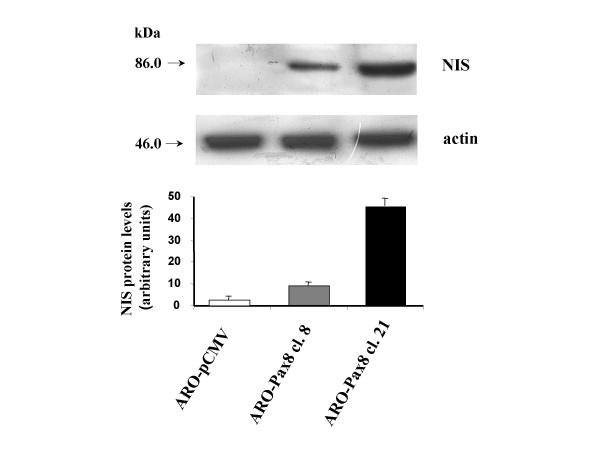
Expression of NIS protein in ARO cells expressing different levels of Pax8 gene. Western Blot analysis was performed as described in Methods. In upper panel/autoradiograph of a representative experiment. In lower panel/the staining intensity is expressed as mean ± SD of values obtained from three different experiments. The data are expressed relative to the control values (cells transfected with the empty vector, ARO-pCMV). Statistic analysis was performed using the one-factor ANOVA, followed by *t *test. *P *< 0.05 was considered statistically significant.

**Figure 4:**
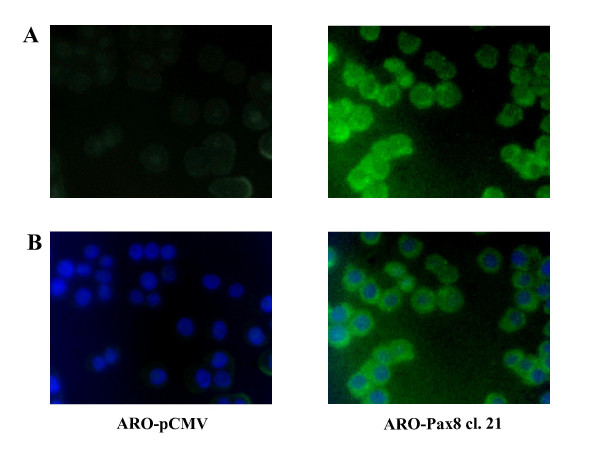
Immunofluorescent localization of NIS in ARO cells. ARO-pCMV and ARO-Pax8 cl. 21 cells were analyzed by immunofluorescence microscopy using rabbit polyclonal anti-NIS and anti-rabbit FITC secondary antibody. A: NIS (green) localization; B. Double immunofluorescence with blue that indicates DAPI nuclear staining and green that indicates the NIS localization. Magnification: 40×.

**Figure 5:**
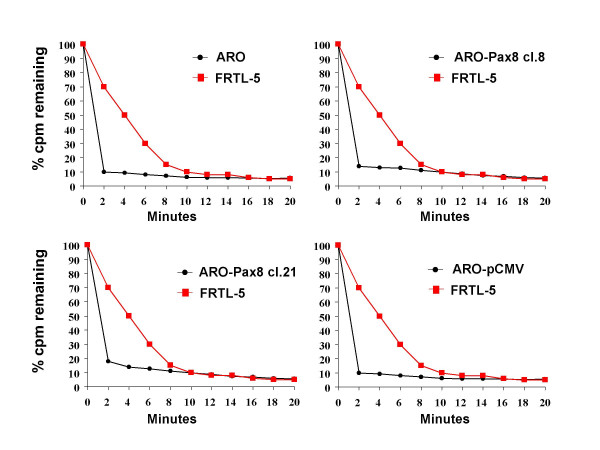
Iodide efflux studies in wild-type and transfected ARO cells. Wild-type and transfected ARO cells (black circles) and FRTL-5 (red squares) grown to 95% confluency in 35-mm diameter plates were incubated in shaking water bath for 60 min at 37°C in HBSS buffer containing 10 μM NaI and 1 μC ^125^I. To evaluate the iodide efflux, the medium was removed and replaced with 1 ml of non-radioactive medium every 2 min. After removal of the last medium (20 min) cells were extracted using 1 ml ethanol (see Methods). The values shown are the counts per min remaining (as percentage of the total) at the indicated times. The experiment has been performed two fold, being no differences in the obtained data.

Goal of the study, however, was to verify the possibility to recover the ability to concentrate the radioiodine. For this reason we evaluated the NIS protein levels by Western blot analysis and its localization by immunofluorescence, together with the ability of ARO-Pax8 cells to uptake the radioiodine. As shown in Fig. 3, NIS protein was detected in both clones and its amount paralleled that of the corresponding transcripts of NIS and Pax8. However, only few amount of the protein reached the plasmamembrane, as detected by immunofluorescence (Fig. 4). Accordingly, a low, but significant rate of radioiodine uptake reappeared in ARO-Pax8 cells with a similar pattern between the two clones and at much lesser extent than normal thyrocytes (Table 2). It was inhibited by perchlorate (10 μM) (Table 2) and not stimulated by activation of the cAMP pathway obtained by incubating the cells with 10 μM of Forskolin (data not shown). A high rate of efflux was also observed, at similar extent in both clones expressing Pax8 and earlier and much higher than that detected in FRTL-5 cells (Fig. 5).

**Figure 6:**
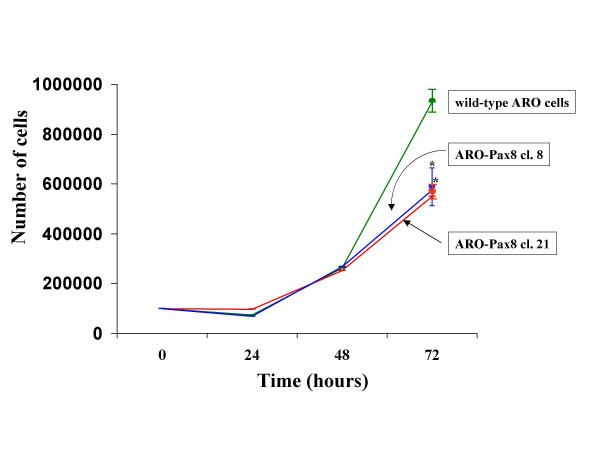
Growth rate of wild-type and transfected ARO cells. 10^5 ^cells were plated in 60 mm dishes. Seven hours were considered as extra time needed from cells to sediment and start to proliferate. Cells were harvested after 24, 48 and 72 hours and the number of cells was determined by Neubauer cell counting chamber. *p value <0.001

Finally, in the ARO-Pax8 clones, we observed a lower growth rate in comparison to ARO non transfected cells (Fig. 6).

## Discussion

A modified gene expression represents the basis for the development and acquisition of the abnormal growth and differentiation characteristics of tumour cells. Since the regulation of the gene expression takes place mainly at transcriptional level, structural or functional alterations of transcription factors, especially those specific for a given tissue, play a key role in the oncogenic process [18]. In thyroid cells, the simultaneous expression of a cohort of tissue-specific transcription factors, including TTF1, TTF2, Pax8 and HEX has been described [19]. Their expression and correct function is essential for the normal development of thyroid gland, and is often altered (reduced or lost) in thyroid tumours, especially in the poorly differentiated histotypes [11]. As a result, thyroid tumour cells present a dedifferentiated phenotype, including a reduced/lost ability to concentrate the radioiodine. This feature represents one of the major determinant of the poor prognosis of less differentiated thyroid carcinomas, since it does not allow the use of radioiodine as diagnostic and therapeutic tool for both tumour remnants or distant metastases. For this reason, attempts to reintroduce such a functional activity in tumour cells have represented the goal of many studies. The cloning and characterization of the protein responsible for iodide uptake, the NIS [20], has opened the way to novel therapeutic strategies based on induction of iodide uptake ability in tumour cells by recovery the expression of the NIS gene. Subsequently, a NIS-based gene therapy approach by using viral vectors has been exploited [4-7]: despite promising results obtained in both in vitro and in vivo experimental models, unresolved issues still remain before proposing such a treatment on humans, mainly a great efflux rate of the administered radioiodine which makes the effective dose of radioisotope (to be administered to destroy the tumour tissue) too high to be side effect free. A parallel approach to restore radioiodine uptake is to induce endogenous NIS expression and correct function by acting on its transcriptional regulation. Various molecules have been challenged, including retinoids, demethylating agents and inhibitors of histone deacetylation [21-25], with preliminary promising effects in various in vitro and in vivo experimental models. However, in poorly differentiated thyroid tumour cells, re-expression of NIS protein is not always accompanied by recovery of its function [26] and, at present, despite their unequivocal activity in vitro, retinoids did not show a sufficient effectiveness in clinical trials on human patients [27]. Probably, alterations of other proteins, including some involved in the intrathyroidal metabolism of iodine or in the correct folding, trafficking, polar localization and function of the NIS, do occur in tumour cells, making not fully effective the simple restoration of NIS expression.

In this work, we explored an alternative way to recover radioiodine concentration ability in thyroid tumour cells. Indeed, we tried to activate the transcriptional machinery which physiologically control NIS gene expression by acting on its promoter, as well as other thyroid specific gene cooperating to express the differentiated thyrocyte phenotype. On this regard, the paired domain-containing protein Pax8 has been characterized for its physiological role in the development of thyroid tissue and maintenance of thyrocyte differentiation phenotype [28,29]. Moreover, reduction/lost of Pax8 expression has been described in thyroid tumour tissues and cell lines [[11], our unpublished observations]. Pax8-binding sites have been described in the promoters of human TPO, Thyroglobulin and recently, also NIS gene [8,9]. In addition, re-expression of Pax8 was associated with the recovery of the NIS, as well as TPO and Tg mRNA expression in a rat thyroid cell line [30]. An effect on Tg and TPO promoter activity had been previously described, in cooperation with TTF-1, also in human thyroid tumour cell lines [31].

Our study demonstrates that thyroid tumour cells expressing Pax8 after stable transfection become able to uptake the radioiodine, even if in absence of a full recovery of NIS protein expression on their plasmamembrane. In addition, other markers of thyroid differentiation were detectable after Pax8 expression, including proteins involved in the intrathyroidal metabolism of iodine such as TPO and pendrin. The expression levels of these thyroid-specific genes were strictly related to the amount of Pax8 expressed in the transfected clones, emphasizing the importance of the levels of this transcription factor for determining a differentiated phenotype [32]. It is of interest also our finding of the recovery of TTF-1 gene expression in the cells with higher levels of Pax8 which suggests a regulatory role for Pax8 at TTF-1 gene promoter level. It may contribute to the phenotypical effects observed and confirms the existence of various levels of cooperation among tissue specific transcription factors [33]. On this regard, few data are available about TTF1 gene promoter, although previous experimental data had suggested the TTF1 gene as a candidate target for regulation by homeobox proteins [34].

However, Pax8 and TTF1 do not determine a full recovery of normal thyrocyte phenotype, as demonstrated by the presence of lower expression levels of the transcripts in comparison with normal thyroid tissue and the absence of detectable levels of the TSH receptor even in the clones overexpressing Pax8, and, more important, the low intracellular retention of radioiodide. In ARO cells, a greater efflux rate had been also observed after overexpression of exogenous NIS gene inserted in a viral vector [35]; similarly, only a faint iodide uptake reappeared in BHP18-21v thyroid tumour cells expressing TTF1 [36]. Moreover, contrasting results have been reported after stimulation of endogenous NIS expression by inhibition of histone deacetylation: by using valproic acid, Fortunati et al. [26] did not obtain a correct localization of the NIS protein in the thyrocyte plasmamembrane, whereas Furuya et al. [25] described a full recovery of iodide uptake also in ARO cells using the histone deacetylase inhibitor depsipeptide. In addition, our observation of the slower growth rate of ARO-Pax8 cells suggest a role for Pax8 in the control of such a function, until now never detected. Altogether, these data indicate that in addition to act on NIS transcription, a more complex strategy including the correct targeting to the plasmamembrane is required to reach the goal of an effective radioiodine intracellular concentration.

## Conclusion

Our finding demonstrate that overexpression of thyroid-specific transcription factors may be a first step to obtain the radioiodine concentration by the transformed thyrocytes, by acting on a pool of differentiation markers including the NIS. Our data, therefore, represent a very promising starting point for further investigations and strategies exploiting this effect of Pax8, eventually in combination of other thyroid-specific transcription factors, for extending radioiodine treatment also to the less differentiated thyroid tumours.

## Competing interests

The author(s) declare that they have not competing interests.

## Pre-publication history

The pre-publication history for this paper can be accessed here:



## Figures and Tables

**Table 1:** Sequences of primer pairs and TaqMan probes

Gene	*Primer forward*	*Primer reverse*	*Probe*
**Pax8**	5'-CAACAGCACCCTGGACGAC-3'	5'-AGGGTGAGTGAGGATCTGCC-3'	5'-CTGACCCCTTCCAACACGCCACTG-3'
**NIS**	5'-CCATCCTGGATGACAACTTGG-3'	5'-AAAAACAGACGATCCTCATTGGT-3'	5'-AGAACTCCCCACTGGAAACAAGAAGCCC-3'
**TPO**	5'-ACGCCTCTGCGAGGTGC-3'	5'-TGCAAATCACCGTCGAGGT-3'	5'-TGCTGATCGGAGGCTTCGCAG-3'
**TSH-R**	5'-CCTTCACCTCACACGGGCT-3'	5'-TGCTCTCATTACACATCAAGGACTC-3'	5'-TTTCTTACCCAAGCCACTGCTGTGCC-3'
**TTF-1**	5'-CCTGTCCCACCTGAACTCCTC-3'	5'-GCCTTTGTGGTTTTTTGTTCC-3'	5'-CGGACTACGGCACCATGTCCTGC-3'
**Pendrin**	5'-CATCAAGACATATCTCAGTTGGACCT-3'	5'-ACAGTTCCATTGCTGCTGGAT-3'	5'-TCTGAGCATGGCCCCCGACG-3'

For Thyroglobulin gene were used primers and probes provided by Applied Biosystems (Assays-on-demand gene products).

**Table 2:** Radioiodide uptake in wild-type and transfected ARO cells

Cell type	Iodide uptake (cpm/well)			
	
		*P*	+ KClO_4_	*P*
ARO wild-type	133 ± 35		78 ± 26	**0.09
ARO pCMV	137 ± 42	*0.9	82 ± 47	**0.2
ARO-PAX8 clone 8	307 ± 43	*0.006	126 ± 27	**0.003
ARO-PAX8 clone 21	257 ± 36	*0.01	104 ± 26	**0.004
FRTL-5	4350 ± 320			
CHO	40 ± 12			

Iodide uptake was analyzed as described in Methods. The FRTL-5 cells and CHO cells were used as positive and negative controls, respectively. Iodide uptake was expressed as the mean ± SD of values obtained from three separate experiments. Statistic analysis was performed using the one-factor ANOVA, followed by *t *test. *P *< 0.05 was considered statistically significant.

* ARO PAX8 clone 8, clone 21 and ARO pCMV *vs *ARO wild-type;

** Uptake with *vs *Uptake without KClO_4_
